# Association between advanced lung cancer inflammation index and acute gouty arthritis in Dalian, China: a cross-sectional study

**DOI:** 10.3389/fnut.2025.1511642

**Published:** 2025-02-14

**Authors:** Ziran Xiu, Zhengnan Gao, Lan Luo

**Affiliations:** ^1^Graduate School, Dalian Medical University, Dalian, China; ^2^Clinical Skills Training Center, Central Hospital of Dalian University of Technology (Dalian Municipal Central Hospital), Dalian, China; ^3^Endocrinology and Metabolism, Central Hospital of Dalian University of Technology (Dalian Municipal Central Hospital), Dalian, China

**Keywords:** acute gouty arthritis, advanced lung cancer inflammation index, cross-sectional study, L-shaped curve, nutrition

## Abstract

**Introduction:**

Acute gouty arthritis (AGA) is an inflammatory joint disease. The advanced lung cancer inflammation index (ALI) evaluates inflammation. This study investigates the association between ALI and AGA.

**Methods:**

We included 652 participants in this cohort study, dividing them into two groups: those with AGA and a control group without AGA. We employed logistic regression to examine the ALI-AGA relationship, using restricted cubic splines (RCS) to assess dose–response relationships, performing subgroup analyses, and conducting interaction tests. K-fold cross-validation was applied for model assessment. Receiver operating characteristic (ROC) curves were applied to visualize and compare the predictive value of ALI and other inflammatory indices for AGA.

**Results:**

Among the 652 participants, the AGA group exhibited significantly lower ALI values compared to the control group. Multivariate logistic regression identified an inverse relationship between ALI and AGA. RCS analysis indicated an L-shaped non-linear relationship between ALI levels and AGA, with inflection points at an ALI of 23.38. Subgroup analyses showed no significant interactions between ALI levels and AGA after stratifying by age, hypertension, coronary heart disease (CHD), and diabetes (DM). The results from the ROC curves indicate that ALI may serve as a better predictor of AGA.

**Conclusion:**

This cross-sectional study found a significant inverse correlation between ALI levels and AGA prevalence. Moreover, the ALI could serve as a more accurate diagnostic tool for AGA and offer a novel approach for further investigating the relationship between inflammation and AGA.

## Introduction

Gout, the most prevalent form of inflammatory arthritis (1), results from the deposition of monosodium urate (MSU) crystals into joint tissues due to systemic hyperuricemia (2). In 2020, approximately 55.8 million people worldwide were affected by gout, representing a 22.5% increase since 1990. The number of males is 3.26 times that of females, and this disparity increases with age, suggesting a higher prevalence of gout in males (3). Gout can present in multiple forms, including acute gout flare (known as acute arthritis), chronic gouty arthritis, tophaceous gout (which involves the formation of tophi), impaired renal function, and urolithiasis (kidney stones) (4). Acute gouty arthritis (AGA) is a common ailment, especially among middle-aged and elderly populations (5). AGA manifests with intense joint pain, redness, warmth, and welling, affecting one or more joints and can evolve into chronic destructive arthropathy (6). Untreated joints can become stiff and malformed, significantly increasing the financial burden of treatment, deteriorating the quality of life, and possibly leading to disability. AGA is triggered by the deposition of MSU, which acts as an endogenous danger signal, leading to significant neutrophil infiltration in the affected joints (7). This influx activates the body’s innate immune response, triggering the release of pro-inflammatory cytokines such as IL-1β, TNF-α, and IL-6 through the NLRP3 inflammasome pathway, which in turn initiates the inflammatory cascade (8–10). These cells, along with other immune cells, secrete immunomodulatory cytokines, causing inflammation and pain (11–13). Moreover, inflammation can lead to the accumulation of adipocytes and insulin resistance through inflammatory mediators like TNF-α and CRP, resulting in alterations in albumin levels and body weight (14). Studies have shown that inflammation reduces albumin levels and body mass index (BMI) (15–18). Thus, a single inflammatory index may not accurately estimate AGA due to these complexities. While previous studies on AGA have often focused on individual inflammatory markers.

The advanced lung cancer inflammation index (ALI), which includes BMI, albumin, and neutrophil to lymphocyte ratio (NLR), was initially developed for lung cancer (19–22), but has since been applied to other cancers including esophageal, pancreatic, and gastric cancers (17, 23–25). Given ALI’s comprehensive role in assessing inflammation, its utility has been extended to studying the prognosis of inflammation-related diseases such as hypertension, heart failure, and coronary artery disease (26–29). The relationship between the ALI and AGA remains unexplored, underscoring the need for further investigation. Given that gout predominantly affects males, this study examines the association between ALI—a comprehensive indicator combining inflammation and nutritional status—and its impact on AGA in males. The study aims to provide robust, flexible modeling to capture complex relationships and offer a more comprehensive understanding of potential variations in AGA.

## Materials and methods

### Study population

**Figure 1:**
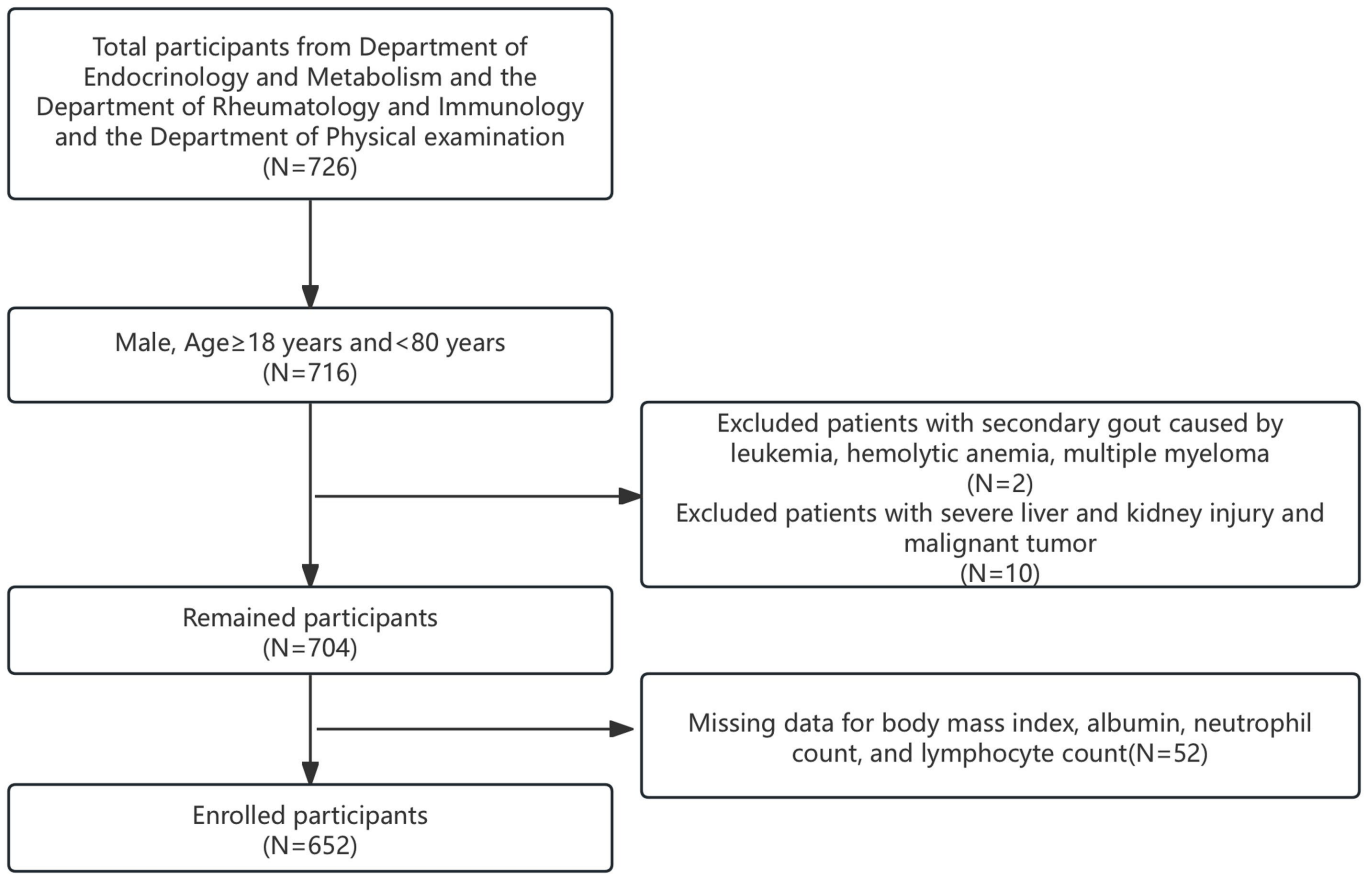
Screening flowchart.

The clinical data of 168 patients diagnosed with primary acute gouty arthritis, as per the 2015 American College of Rheumatology criteria (30), were retrospectively analyzed. These patients were admitted to the Department of Endocrinology and Rheumatology between 2014 and 2024. Concurrently, 558 individuals from the physical examination department served as healthy controls, totaling 726 participants. Initially, 716 male participants aged 18 to 80 years were selected. From this group, 12 individuals were excluded: two with secondary gout due to leukemia, hemolytic anemia, or multiple myeloma, and 10 with severe liver or kidney damage and malignant tumors. Furthermore, 52 participants lacking complete data on BMI, albumin levels, and NLR were also excluded. Ultimately, 652 participants were included in this cohort study and were divided into two groups: those with acute gouty arthritis (AGA group) and controls (control group), as illustrated in Figure 1.

### Calculation of inflammatory indices

Advanced lung cancer inflammation index was calculated with the formula: BMI (kg/m^2^) × albumin (g/dL)/NLR.

Neutrophil to lymphocyte ratio was calculated with the formula: neutrophil counts (10^9^/L)/lymphocyte counts (10^9^/L).

Systemic inflammation response index (SIRI) was calculated with the formula: neutrophil counts (10^9^/L) × monocyte counts (10^9^/L)/lymphocyte counts (10^9^/L).

Systemic immune inflammation index (SII) was calculated with the formula: neutrophil counts (10^9^/L) × platelet counts (10^9^/L)/lymphocyte counts (10^9^/L).

Pan-immune-inflammation value (PIV) was calculated with the formula: [neutrophil count (10^9^/L) × platelet count (10^9^/L) × monocyte count (10^9^/L)]/lymphocyte count (10^9^/L).

Hemoglobin, albumin, lymphocyte, and platelet (HALP) score was calculated with the formula: hemoglobin (g/L) × albumin (g/L) × lymphocyte count (10^9^/L)/platelet count (10^9^/L).

### Covariates

Age, sex, smoking status, alcohol consumption, and the history of hypertension, diabetes (DM), and coronary heart disease (CHD) were self-reported by participants. Self-reported data is a common practice in epidemiological studies (31). Upon admission, body weight, height, systolic blood pressure (SBP), and diastolic blood pressure (DBP) were measured. The BMI was calculated as weight divided by height squared. Laboratory measurements included triglycerides (TG), high-density lipoprotein cholesterol (HDL), low-density lipoprotein cholesterol (LDL), glucose (Glu), creatinine (Cr), uric acid (UA), albumin, neutrophil counts, and lymphocyte counts, monocyte count, platelet count, and hemoglobin.

### Statistical analysis

Normality tests were performed on all data, and the Mann–Whitney *U*-test was applied to variables that did not follow a normal distribution. The results are presented as median values with interquartile ranges (Q25, Q75). Frequency and percentage (*n*%) were used to characterize categorical variables, and the chi-square test was used to determine group differences. A multifactorial logistic regression was used to explore the relationship between ALI and AGA. In the absence of predefined cut-off points, the interquartile method was used for analysis. Three analytical models were developed: Model 1 (unadjusted), Model 2 (adjusted for age), and Model 3 (adjusted for multiple variables including age, HDL, Glu, Cr, UA, Hypertension). The dose–response curves between ALI and AGA were analyzed using 4-knot restricted cubic splines (RCS). Subgroup analyses were conducted to explore potential modifications by covariates such as age, hypertension, CHD, and DM might modify the relationship between ALI and AGA, considering interactions significant at *p* < 0.05. The predictive capacity of ALI for AGA was assessed using ROC curve analysis, obtaining area under the curve (AUC), sensitivity, and specificity values. As part of the model validation process, we employed *k*-fold cross-validation (*k* = 10) to rigorously assess the predictive performance of our multivariate logistic regression model. All statistical analyses were performed using EmpowerStats (version 4.2) and R software (version 4.3.2), considering a two-sided *p*-value of less than 0.05 as statistically significant.

## Results

### Baseline characteristics

We initially extracted data for 726 participants from those with AGA attending endocrinology and rheumatology departments and a non-gout population visiting the physical examination department. After applying inclusion and exclusion criteria, 652 participants were deemed eligible. The prevalence of AGA in the study population was 24.39%. The mean age of all included individuals was 55.00 years. As detailed in Table 1, the AGA group exhibited higher values for BMI, Glu, ALT, Cr, UA, neutrophils, the prevalence of hypertension, NLR, SII, SIRI, and PIV compared to the control group. Conversely, the AGA group showed significantly lower levels of age, TC, HDL, albumin, lymphocytes, hemoglobin and HALP relative to the control group. The AGA group also had significantly lower values for the ALI compared to controls. The overall average ALI in our study was 56.67, as shown in Table 1.

**Table 1:** Baseline characteristics.

Variable	Overall	Control	Acute gouty arthritis	*P*-value
	*N* = 652	*N* = 493	*N* = 159	
ALI (mean)	56.67 (38.72, 76.93)	63.46 (46.55, 82.45)	34.06 (19.15, 53.24)	<0.05
Age (years)	55.00(45.00, 63.00)	56.00 (47.00, 64.00)	53.00 (40.00, 62.50)	<0.05
SBP (mmHg)	130 (122, 140)	130 (123, 140)	130 (120, 140)	0.07
DBP (mmHg)	83 (78, 90)	83 (78, 91)	80.00 (78, 90)	0.12
BMI (Kg/m^2^)	25.10 (23.00, 27.70)	24.80 (22.80, 27.30)	26.40 (24.10, 29.25)	<0.05
TG (mmol/L)	1.36 (0.96, 1.97)	1.34 (0.95, 1.95)	1.37 (0.98, 2.00)	0.48
HDL (mmol/L)	1.00 (0.87, 1.16)	1.03 (0.90, 1.21)	0.91 (0.76, 1.05)	<0.05
LDL (mmol/L)	2.95 (2.35, 3.48)	2.98 (2.39, 3.51)	2.88 (2.23, 3.39)	0.20
Glu (mmol/L)	5.17 (4.70, 5.76)	5.09 (4.68, 5.62)	5.44 (4.88, 6.11)	<0.05
Cr (μmol/L)	73.60 (66.05, 82.80)	71.55 (65.10, 78.23)	86.35 (73.70, 103.15)	<0.05
UA (μmol/L)	390.00 (329.50, 455.00)	374.50 (322.00, 433.00)	458.00 (372.00, 547.00)	<0.05
Albumin (g/dL)	4.30 (4.04, 4.52)	4.36 (4.11, 4.56)	4.07 (3.82, 4.41)	<0.05
Neutrophils (×10^9^/L)	3.54 (2.8, 4.62)	3.25 (2.66, 4.02)	5.40 (4.05, 7.26)	<0.05
Lymphocytes (×10^9^/L)	1.85 (1.46, 2.30)	1.90 (1.50, 2.31)	1.72 (1.23, 2.21)	<0.05
Monocyte (×10^9^/L)	0.38 (0.30, 0.46)	0.38 (0.30,0 0.47)	0.38 (0.30, 0.43)	0.22
Platelet (×10^9^/L)	219.00 (177.00, 264.25)	216.00 (178.00, 262.00)	226.00 (176.00, 276.00)	0.19
Hemoglobin (g/L)	136.00 (124.00, 148.00)	140.00 (129.00, 150.00)	128.00 (113.00, 136.00)	<0.05
Hypertension (%)				<0.05
0	486 (74.54%)	399 (80.93%)	87 (54.72%)	
1	166 (25.46%)	94 (19.07%)	72 (45.28%)	
CHD (%)				0.22
0	636 (97.55%)	483 (97.97%)	153 (96.23%)	
1	16 (2.45%)	10 (2.03%)	6 (3.77%)	
DM (%)				0.55
0	594 (91.10%)	451 (91.48%)	143 (89.94%)	
1	58 (8.90%)	42 (8.52%)	16 (10.06%)	
Drinking alcohol (%)				0.53
0	388 (59.51%)	290 (58.82%)	98 (61.64%)	
1	264 (40.49%)	203 (41.18%)	61 (38.36%)	
Smoking (%)				0.15
0	345 (52.91%)	253 (51.32%)	92 (57.86%)	
1	307 (47.09%)	240 (48.68%)	67 (42.14%)	
NLR	1.89 (1.43, 2.70)	1.72 (1.34, 2.19)	3.20 (2.10, 5.33)	<0.05
SII	408.92 (290.66, 612.74)	363.09 (268.21, 500.19)	691.93 (443.84, 1239.43)	<0.05
SIRI	0.74 (0.51, 1.07)	0.65 (0.47, 0.90)	1.16 (0.75, 1.81)	<0.05
PIV	150.85 (103.16, 246.74)	141.05 (93.92, 198.18)	258.11 (148.92, 446.24)	<0.05
HALP	48.40 (34.49, 66.94)	50.76 (38.35, 69.45)	37.54 (22.80, 56.59)	<0.05

For continuous variables, the median (Q25, Q75) was calculated and compared using the Mann–Whitney *U*-test. For categorical variables, percentages were used and the *p*-value was calculated using the chi-square test. ALI, advanced lung cancer inflammation index; SBP, systolic blood pressure; DBP, diastolic blood pressure; BMI, body mass index; TG, triglycerides; HDL, high-density lipoprotein; LDL, low-density lipoprotein; Glu, glucose; Cr, creatinine; UA, uric acid; CHD, coronary heart disease; DM, diabetes mellitus; NLR, neutrophil-to-lymphocyte ratio; SII, systemic immune inflammation index; SIRI, systemic inflammation response index; PIV, pan-immune-inflammation value; HALP, hemoglobin, albumin, lymphocyte, and platelet.

### Association between ALI and AGA

To investigate the relationship between ALI and AGA, we developed three multivariate logistic regression models. As detailed in Table 2, the potential confounding factors adjusted in each model were presented in the method section. The odds ratios (ORs) of ALI were 0.96 (95%CI, 0.95–0.97), 0.95 (95% CI, 0.94–0.96), and 0.95 (95% CI, 0.94–0.97), respectively, in Model 1 (no adjustment), Model 2 (adjusted for age), and Model 3 (adjusted for age, HDL, Glu, Cr, UA, Hypertension). To verify the accuracy of our results, we performed a sensitivity analysis examining the relationship between different quartile groups of ALI. In model 3, the ORs were 0.16 (95%CI, 0.08–0.33) for Q2, 0.09 (95%CI, 0.04–0.20) for Q3, and 0.04 (95%CI, 0.02–0.10) for Q4 compared to Q1. A progressive strengthening of these associations across quartiles was observed, indicating a significant positive trend (*P* for trend < 0.05). For assess the model’s predictive performance and generalizability, we used a *K*-fold cross-validation (*k* = 10). In 10-fold cross-validation for the three models, Model 1 achieved a ROC of 0.786, with a sensitivity of 95.53% and a specificity of 38.33%. Model 2 showed an improvement with a ROC of 0.819, sensitivity of 94.94%, and specificity of 43.96%. Model 3 demonstrated the highest ROC at 0.864, though it had a lower sensitivity of 51.86%, it achieved a high specificity of 95.52%.

**Table 2:** Relationship between ALI and AGA.

	Model 1		Model 2		Model 3	
	OR (95%CI)	*P*-value	OR (95%CI)	*P*-value	OR (95%CI)	*P*-value
ALI	0.96 (0.95,0.97)	<0.05	0.95 (0.94,0.96)	<0.05	0.95 (0.94,0.96)	<0.05
Q1	1.0 (Ref)		1.0 (Ref)		1.0 (Ref)	
Q2	0.23 (0.14,0.37)	<0.05	0.19 (0.11,0.31)	<0.05	0.16 (0.08,0.31)	<0.05
Q3	0.11 (0.06,0.20)	<0.05	0.09 (0.05,0.16)	<0.05	0.09 (0.04,0.20)	<0.05
Q4	0.07 (0.04,0.13)	<0.05	0.05 (0.03,0.10)	<0.05	0.04 (0.02,0.10)	<0.05
*P*-trend	<0.05		<0.05		<0.05	

Q1 3.99–38.73, Q2 38.74–56.76, Q3 56.77–76.99, Q4 77.00–283.40.

Model 1: no adjustment.

Model 2: adjusted for age.

Model 3: adjusted for age, HDL, Glu, Cr, UA, hypertension.

### RCS curves fitting

**Figure 2:**
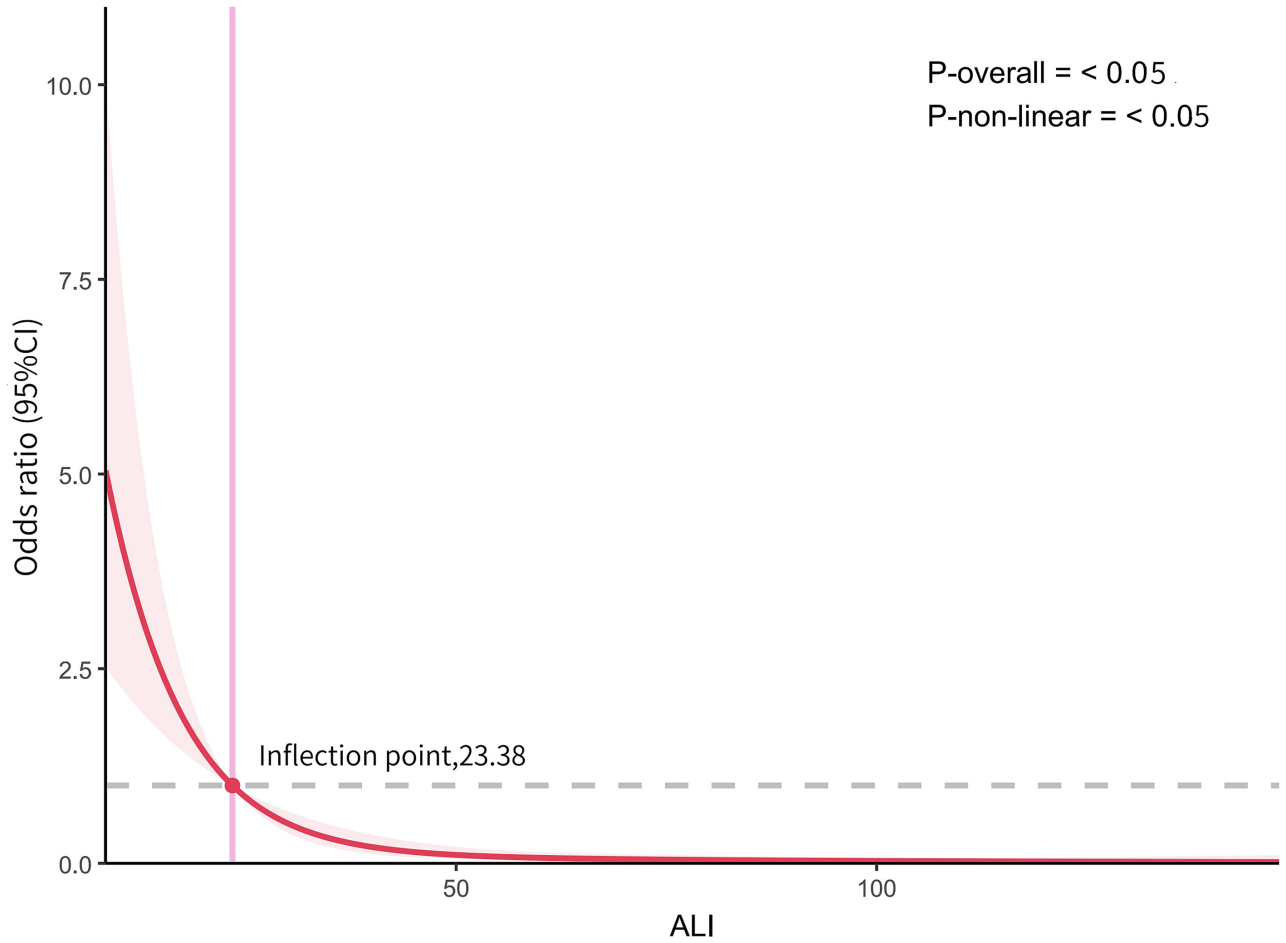
The dose–response relationship between ALI and AGA. Graphs show OR for AGA adjusted for age, HDL, Glu, Cr, UA, Hypertension. Data were fitted by multivariate logistic regression models. Solid lines indicate OR, and shadow shapes indicate 95% CIs.

Using 4-knots RCS curves fitting, we analyzed the dose–response relationship between ALI and AGA. As detailed in Figure 2, following adjustments for multiple confounding variables, the RCS analysis indicated that an L-shaped non-linear relationship existed between ALI levels and AGA, with statistical significance (*p* < 0.05). The study identified an inflection point for AGA at 23.38. The findings from the RCS fitting are consistent with those obtained from multivariate regression analysis.

### Subgroup analysis

**Figure 3:**
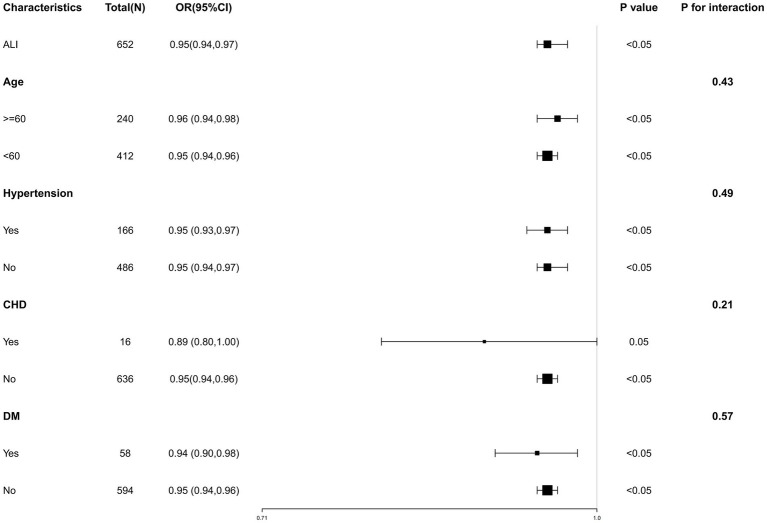
Subgroup analysis for the association of ALI and AGA. Subgroup analysis of the association between ALI and AGA, stratified by age, hypertension, CHD, and DM. Each square in the forest plot represents an estimated Odds Ratio (OR) for a specific subgroup. The size of the square is usually proportional to the statistical weight or sample size of that estimate. “P for interaction” tests whether there is any interaction between the subgroups.

We performed a subgroup analysis based on age, hypertension, CHD and DM to assess the robustness of our results across different groups. The results of the subgroup analysis were displayed in Figure 3, showing consistency across all subgroups. We fully adjusted the model for age, HDL, Glu, Cr, UA, hypertension. No significant interactions were observed between AGA and ALI level in subgroup analysis, with all *p*-values for interaction exceeding 0.05.

### ROC analysis of ALI and other inflammatory indices for predicting AGA

**Figure 4:**
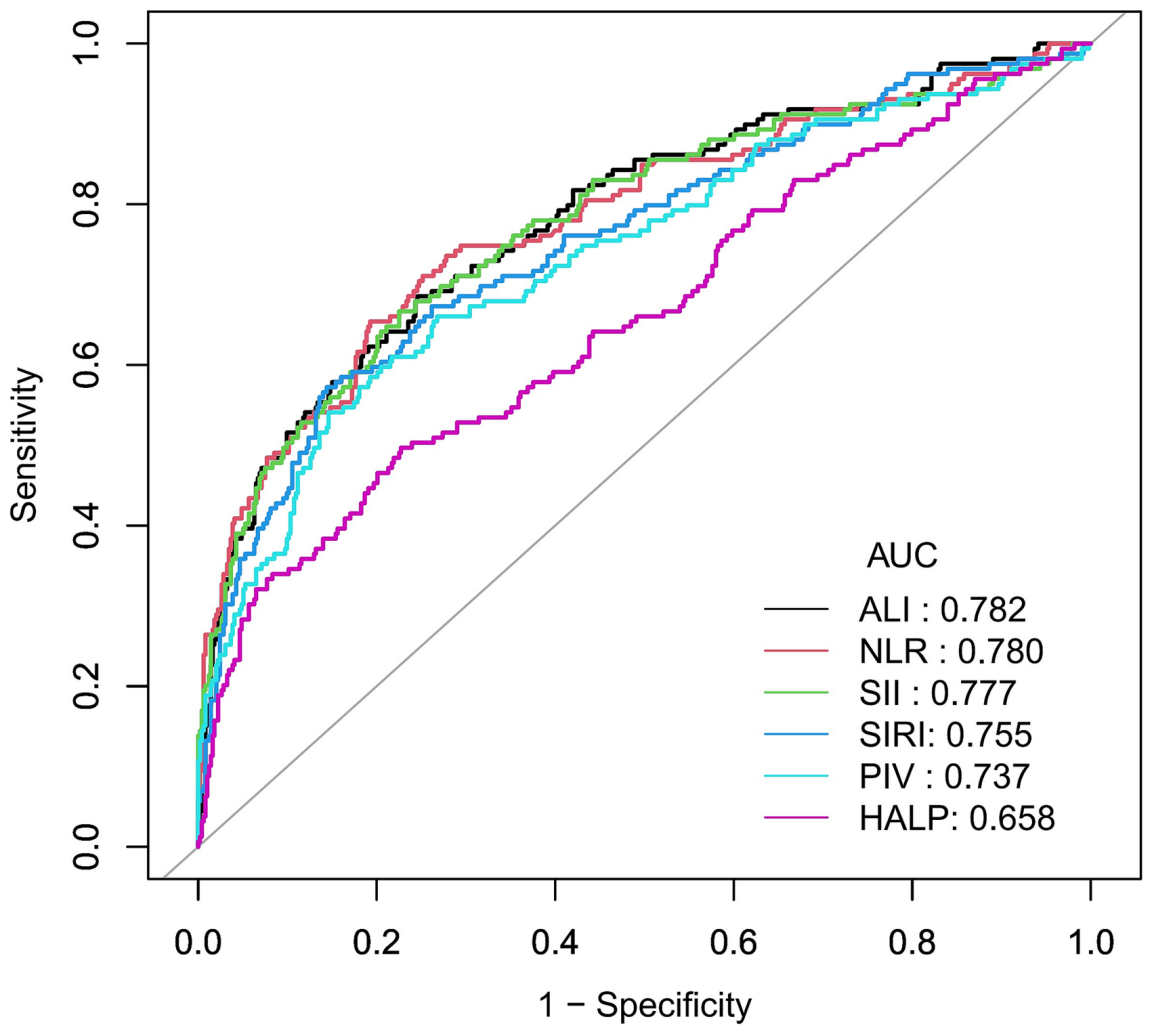
Receiver operating characteristic (ROC) curve for AGA prevalence. Predicting AGA using Receiver Operating Characteristic (ROC) curve. Comparison of area under curve (AUC) value between ALI, NLR, SII, SIRI, PIV, and HALP. The black curve represents ALI, the red curve represents the NLR, the green curve represents SII, the blue curve represents SIRI, the teal curve represents PIV, and the pink curve represents HALP.

To further investigate the diagnostic performance of ALI and other inflammatory indices in predicting AGA, we conducted ROC curve analyses and assessed the diagnostic potential using the AUC values and optimal cut-off values. ALI, along with five other inflammatory indices, demonstrated the ability to identify AGA. The AUC value of ALI (0.782) was significantly higher than those of NLR (0.780), SII (0.777), SIRI (0.755), PIV (0.737), and HALP (0.658). The optimal cut-off values for ALI, NLR, SII, SIRI, PIV, and HALP were 46.28, 2.39, 516.15, 1.07, 249.86, and 37.02, respectively. These results suggest that ALI may be superior to NLR, SII, SIRI, PIV, and HALP in diagnosing AGA (Table 3 and Figure 4).

**Table 3:** Receiver operating characteristic (ROC) curves for the prediction of AGA by inflammatory indices.

	AUC	95%CI	Cut-off value	Specificity (%)	Sensitivity (%)	Youden index
ALI	0.782	0.74–0.83	46.28	75.46	68.55	0.44
NLR	0.780	0.74–0.83	2.39	80.73	65.41	0.46
SII	0.777	0.73–0.82	516.15	77.48	66.67	0.44
SIRI	0.755	0.71–0.80	1.07	85.60	57.23	0.43
PIV	0.737	0.69–0.79	249.86	85.40	54.09	0.40
HALP	0.658	0.61–0.71	37.02	77.28	49.69	0.27

AUC, area under the curve; ALI, advanced lung cancer inflammation index; NLR, neutrophil-to-lymphocyte ratio; SII, systemic immune inflammation index; SIRI, systemic inflammation response index; PIV, pan-immune-inflammation value; HALP, hemoglobin; albumin, lymphocyte, and platelet.

## Discussion

In this cross-sectional investigation, we utilized ALI as an indicator of inflammation in relation to AGA. Our findings demonstrated a strong inverse relationship between increased levels of ALI and reduced risk of AGA, persisting even after adjusting for various confounders. The negative correlation became more pronounced across quartiles, exhibiting a significant upward trend (*P* for trend <0.05). RCS analyses indicated an L-shaped non-linear association between ALI and AGA, where levels decline to a certain point and then stabilize, with the turning point identified at 23.38. This relationship was consistent across different demographic and health conditions including age, hypertension, CHD, and DM. These findings support the association of ALI with AGA risk. This research marks the first exploration of the potential connections between ALI and AGA in the Dalian area of China.

This study demonstrates an L-shaped non-linear relationship between ALI and AGA. To investigate the reasons, consider three aspects. Firstly, NLR reflects the dynamic interaction between innate (neutrophil) and adaptive cellular immune responses (lymphocyte) during disease and various pathological states. Monosodium urate crystals can directly recruit neutrophils to accumulate in joints during acute gout attacks (32, 33). Neutrophils phagocytose the crystals, produce reactive oxygen species (ROS) via the NADPH enzyme, and released a variety of neutrophil chemoattractants. Recruited and activated neutrophils promote the massive release of inflammatory mediators and aggravate joint inflammation (34). Systemic inflammation typically results in increased neutrophils and decreased lymphocytes (33). It has been suggested that lymphocyte counts are low in patients with gouty arthritis and play a crucial role in the resolution and repair of inflammation (35). The above mechanism may explain the change of NLR in acute gouty arthritis and is also consistent with previous studies (36, 37). Secondly, albumin is the most commonly used indicator for nutritional assessment. During acute inflammation, inflammatory factors inhibit protein synthesis. Crevel et al. (38) noted that elevations proinflammatory cytokines suppress albumin production during cachexia. In comparison, the high albumin group exhibited significantly lower levels of TNF and CRP compared to the low albumin group (39). In the present study, we noted a decrease in albumin levels in the AGA group relative to the control group, consistent with the above. Finally, because high BMI is a well-established risk factor for gout, it is critical to take this factor into account when evaluating the association. Research indicates that greater body mass index increases risk of gout (40, 41). Therefore, ALI which integrates the NLR, albumin and BMI, provides a more comprehensive reflection of inflammation in AGA. However, further research is required to elucidate this association fully. Notably, the inflection point for AGA was identified at 23.38. The study suggested that exceeding this inflection point does not increase the risk of AGA when ALI is elevated. This indicates that clinically, caution is warranted for patients with ALI values below this threshold, as maintaining specific ALI levels may reduce the risk of AGA. These results suggest that keeping ALI levels within a specific range, such as managing body weight and maintaining normal albumin levels, is essential in clinical settings for managing AGA patients. The findings are consistent across different subgroups stratified by age, hypertension, CHD, and DM.

Recent studies have aimed to identify the most effective inflammatory markers for predicting AGA in Chinese populations. The blood inflammation response index is a novel, non-specific marker that incorporates the NLR, SII, and SIRI. These indices offer a more comprehensive assessment of systemic inflammation. The study found that these indices are significantly associated with the severity of inflammation and outcomes in gouty arthritis (37). In this study, we considered the PIV and HALP when comparing the predictive performance of the ALI with other indices. PIV is a novel biomarker that assesses both immune and inflammatory status in patients (42). Additionally, the HALP score, which integrates hemoglobin, albumin, lymphocyte count, and platelet count, provides a comprehensive evaluation of both the inflammatory response and nutritional status (43). Although these markers have not been extensively studied for predicting AGA, their potential in this area warrants further exploration. In our study, we compared the predictive performance of ALI with NLR, SII, SIRI, PIV, and HALP. We found that ALI demonstrated the strongest predictive power for AGA in men, as indicated by its highest AUC value. Thus, our study makes a significant contribution to the existing literature on this topic.

Our findings have potential clinical implications. While traditional clinical criteria and serum uric acid levels remain essential for diagnosing and managing AGA, the ALI may provide additional insights, particularly in complex cases characterized by systemic inflammation. First, utilizing ALI for risk stratification could improve our understanding of how different subgroups respond to treatment, potentially leading to more personalized therapeutic approaches. Moreover, ALI could serve as a valuable biomarker for monitoring systemic inflammation dynamics in AGA patients. Finally, elevated ALI scores may predict flare-ups or disease deterioration, thereby facilitating the implementation of appropriate therapeutic interventions.

Our study offers several strengths and acknowledges certain limitations. Strengths include the use of a geographically consistent sample of AGA patients from Dalian, China, which minimizes sample bias and enhances representativeness. To improve the external validity of our results, we compared key variables in our sample—such as age, BMI, and the prevalence of hypertension and diabetes—with data from the following studies: According to the study Trends of Gout Disease Burden in China from 1990 to 2019 and Forecast for the Next 10 Years (44), the peak age for male patients with gout is between 45 and 69 years. In this study, the median age of male patients with acute gout was 53 years (range 40–62.5 years), which corresponds to the typical age of gout attacks. A study by Aune et al. (41) demonstrated that higher BMI increases the risk of gout. The relative risks for gout were 1.78, 2.67, 3.62, and 4.64 for BMIs of 25, 30, 35, and 40 kg/m^2^, respectively, compared to a BMI of 20 kg/m^2^. Choi et al. (45) showed that gout patients with a BMI of 25–29.9 kg/m^2^ had a 1.95 times higher risk of gout than those with a BMI of 21–22.9 kg/m^2^. A study conducted by North Sichuan Hospital on the influence of inflammatory factors on acute pain (37) found that the average BMI of acute gout patients was 25.79 ± 3.40. In our study, the BMI was 26.4 (range 24.10–29.25), which is similar to these findings. According to the Chinese Guidelines for Diagnosis and Treatment of Hyperuricemia and (46), 47.2–77.75% of patients with hyperuricemia and gout also have hypertension, and 12.2–26.9% have diabetes (46). In our study, the prevalence of hypertension and diabetes among patients with acute gout was 45.28 and 10.06%, respectively, which is similar to the results reported in these studies. Additionally, a study by North Sichuan Hospital on the influence of inflammatory factors on acute gout (37) found that the prevalence of diabetes was 8.6%, which is comparable to our findings. Through these comparisons, we believe that our sample is representative of the general population, thereby enhancing the external validity of our research results. Meanwhile, we employed multiple logistic regression analysis, stratified analysis, and interaction analysis to account for various confounding factors, thereby bolstering the reliability of our results. Finally, we have reviewed comparable studies and found that our sample size is neither excessively large nor insufficiently small (36, 37, 47), ensuring the reliability of our findings. Limitations of this study are notable: (1) It did not consider significant AGA influencing factors such as diet and medication, limiting our ability to fully address these potential confounders. (2) In this study, the collection of medical history—specifically hypertension, diabetes, and coronary heart disease—was based on self-reported data from patients, which may introduce potential bias and affect the reliability of the study’s findings. (3) Compared to cohort studies, our cross-sectional study design restricts the capacity to investigate etiology and prognostic factors, necessitating further research with more comprehensive clinical data and additional cohort studies.

## Conclusion

Our research demonstrated a significant association between higher ALI levels and a reduced risk of AGA. The data suggested that ALI and AGA exhibit an L-shaped non-linear relationship, and ALI may serve as a more accurate predictor of AGA. The study highlights the potential utility of ALI in monitoring both the inflammatory and nutritional status of individuals with AGA.

## Data Availability

The raw data supporting the conclusions of this article will be made available by the authors, without undue reservation.
